# Why Do Hubs in the Yeast Protein Interaction Network Tend To Be Essential: Reexamining the Connection between the Network Topology and Essentiality

**DOI:** 10.1371/journal.pcbi.1000140

**Published:** 2008-08-01

**Authors:** Elena Zotenko, Julian Mestre, Dianne P. O'Leary, Teresa M. Przytycka

**Affiliations:** 1 Max-Planck Institute for Informatics, Saarbruecken, Germany; 2 Department of Computer Science, University of Maryland, College Park, Maryland, United States of America; 3 Institute for Advanced Computer Studies, University of Maryland, College Park, Maryland, United States of America; 4 National Center of Biotechnology Information, National Library of Medicine, National Institutes of Health, Bethesda, Maryland, United States of America; Columbia University, United States of America

## Abstract

The *centrality-lethality rule*, which notes that high-degree nodes in a protein interaction network tend to correspond to proteins that are essential, suggests that the topological prominence of a protein in a protein interaction network may be a good predictor of its biological importance. Even though the correlation between degree and essentiality was confirmed by many independent studies, the reason for this correlation remains illusive. Several hypotheses about putative connections between essentiality of hubs and the topology of protein–protein interaction networks have been proposed, but as we demonstrate, these explanations are not supported by the properties of protein interaction networks. To identify the main topological determinant of essentiality and to provide a biological explanation for the connection between the network topology and essentiality, we performed a rigorous analysis of six variants of the genomewide protein interaction network for *Saccharomyces cerevisiae* obtained using different techniques. We demonstrated that the majority of hubs are essential due to their involvement in Essential Complex Biological Modules, a group of densely connected proteins with shared biological function that are enriched in essential proteins. Moreover, we rejected two previously proposed explanations for the centrality-lethality rule, one relating the essentiality of hubs to their role in the overall network connectivity and another relying on the recently published essential protein interactions model.

## Author Summary

Analysis of protein interaction networks in the budding yeast *Saccharomyces cerevisiae* has revealed that a small number of proteins, the so-called hubs, interact with a disproportionately large number of other proteins. Furthermore, many hub proteins have been shown to be essential for survival of the cell—that is, in optimal conditions, yeast cannot grow and multiply without them. This relation between essentiality and the number of neighbors in the protein–protein interaction network has been termed the *centrality-lethality rule*. However, why are such hubs essential? Jeong and colleagues [1] suggested that overrepresentation of essential proteins among high-degree nodes can be attributed to the central role that hubs play in mediating interactions among numerous, less connected proteins. Another view, proposed by He and Zhang, suggested that that the majority of proteins are essential due to their involvement in one or more *essential protein–protein interactions* that are distributed uniformly at random along the network edges [2]. We find that none of the above reasons determines essentiality. Instead, the majority of hubs are essential due to their involvement in Essential Complex Biological Modules, a group of densely connected proteins with shared biological function that are enriched in essential proteins. This study sheds new light on the topological complexity of protein interaction networks.

## Introduction

An intriguing question in the analysis of biological networks is whether biological characteristics of a protein, such as essentiality, can be explained by its placement in the network, i.e., whether topological prominence implies biological importance. One of the first connections between the two in the context of a protein interaction network, the so-called *centrality-lethality rule*, was observed by Jeong and colleagues [3], who demonstrated that high-degree nodes or *hubs* in a protein interaction network of *Saccharomyces cerevisiae* contain more essential proteins than would be expected by chance. Since then the correlation between degree and essentiality was confirmed by other studies [4]–[7], but until recently there was no systematic attempt to examine the reasons for this correlation. In particular, what is the main topological determinant of essentiality? Is it the number of immediate neighbors or some other, more global topological property that essential proteins may have in a protein interaction network?

Jeong and colleagues [3] suggested that overrepresentation of essential proteins among high-degree nodes can be attributed to the central role that hubs play in mediating interactions among numerous, less connected proteins. Indeed, the removal of hubs disrupts the connectivity of the network, as measured by the network diameter or the size of the largest connected component, more than the removal of an equivalent number of random nodes [3],[8]. Therefore, under the assumption that an organism's function depends on the connectivity among various parts of its interactome, hubs would be predominantly essential because they play a central role in maintaining this connectivity.

Recently, He and colleagues challenged the hypothesis of essentiality being a function of a global network structure and proposed that the majority of proteins are essential due to their involvement in one or more *essential protein–protein interactions* that are distributed uniformly at random along the network edges [9]. Under this hypothesis, hubs are proposed to be predominantly essential because they are involved in more interactions and thus are more likely to be involved in one which is essential.

In this work we carefully evaluate each of the proposed explanations for the centrality-lethality rule. Recently several hypotheses that linked structural properties of protein interaction networks to biological phenomena have come under scrutiny, with the main concern being that the observed properties are due to experimental artifacts and/or other biases present in the networks and as such lack any biological implication. To limit the impact of such biases on the results reported in our study we use six variants of the genomewide protein interaction network for *Saccharomyces cerevisiae* compiled from diverse sources of interaction evidence [10]–[15].

To assess whether the essentiality of hubs is related to their role in maintaining network connectivity we performed two tests. First, if this were the case, then we would expect essential hubs to be more important for maintaining network connectivity than nonessential hubs. We found that this is not the case. Next, in addition to node degree, we consider several other measures of topological prominence, and we demonstrate that some of them are better predictors of the role that a node plays in network connectivity than node degree. Thus, if essentiality were related to maintaining network connectivity, then one would expect essentiality to be better correlated with these centrality measures than with the node degree. However, we found that node degree is a better predictor of essentiality than any other measure tested.

To reject the essential protein interaction model [9], we used a hypothesis testing approach. Namely, we observed that this model implies that the probability that a protein is essential is independent of the probability that another noninteracting protein is essential. However, in the tested networks the essentiality of noninteracting proteins that share interaction partners is correlated. Thus, we reject the independence assumption and, as a result, the essential protein interaction model with high confidence.

Motivated by our findings we propose an alternative explanation for the centrality-lethality rule. Our explanation draws on a growing realization that phenotypic effect of gene-knockout experiments is a function of a group of functionally related genes, such as genes whose gene products are members of the same multiprotein complex [16]. It is well known that densely connected subnetworks are enriched in proteins that share biological function. Therefore, one would expect that dense subnetworks of protein interaction networks should be either enriched or depleted in essential proteins. Indeed, Hart and colleagues observed that essential proteins are not distributed evenly among the set of automatically indentified multiprotein complexes [17]. In this work we observe that the same phenomenon holds for potentially larger groups of densely connected and functionally related proteins, which we call COmplex BIological Modules (or COBIMs). We demonstrate that due to the uneven distribution of essential proteins among COBIMs the majority of the essential proteins lie in those COBIMs that are enriched in essential proteins, which we call Essential COmplex BIological Modules (or ECOBIMs).

By the very definition, ECOBIMs contain, relative to their size, more essential nodes than a random group of proteins of the same size. But what fraction of all essential hubs are members of such ECOBIMs? How does this number relate to what is expected by chance? In fact, how does the enrichment of hubs that are members/nonmembers of ECOBIMs in essential proteins relate to the enrichment values expected by chance under a suitable randomization protocol? We propose that membership in ECOBIMs largely accounts for the enrichment of hubs in essential proteins. In support of this hypothesis, we found that the fraction of essential proteins among non-ECOBIM hubs is, depending on the network, only 13–35%, which is almost as low as the network average. Furthermore the essentiality of nodes that are not members of ECOBIMs is only weakly correlated with their degree. Finally, using a randomization experiment we demonstrated that these properties are characteristic of the protein interaction network and are unlikely in a corresponding randomized network.

## Results

### Our Study Uses Six Protein Interaction Networks

Our source of protein interaction data for the yeast *Saccharomyces cerevisiae* is numerous small-scale studies and seven high-throughput experiments [15], [18]–[23]. Interactions reported in targeted studies are believed to be biologically relevant as they are usually subjected to a variety of validation methods. Recently, Reguly et al. [11] curated about 30,000 literature abstracts to compile a network of protein interactions reported in small-scale experiments. We refer to this network as the *LC network* (Literature Curated network).

It was suggested that the centrality-lethality phenomenon is an artifact of a possible bias present in the networks mainly derived from small-scale experiments [24]. Namely, essential proteins are the focus of more studies and therefore tend to have a higher degree in these networks. Therefore, to complement the LC network, we included in our study two networks that contain interactions reported in both small-scale studies and high-throughput experiments. The *DIP CORE network* is derived from the pool of protein interactions deposited in the DIP database using a computational method of Deane et al. [10] that recruits evolutionary information to filter out unreliable interactions. The *HC network* (the High Confidence network) recently published by Batada et al. [12] is derived by intersecting small-scale data with the above-mentioned seven high-throughput datasets. More specifically, an interaction is included in the final network only if it was independently reported at least twice.

We also include two networks derived solely from high-throughput experimental data. The *Y2H network* is obtained from the genomewide yeast-two-hybrid interaction screen of Ito et al. [15] and contains high-confidence interactions that were experimentally detected at least three times. Recently, Collins et al. [13] published a statistical scoring scheme that maps raw complex purification experimental data to interaction confidence scores. The authors applied their method to raw purification data from two recent genomewide complex purification experiments [22],[23]. We refer to a network that contains all interactions with a confidence score above a certain threshold as the *TAP-MS network*.

Finally, we include a network of interactions predicted in silico using the computational approach of Jansen et al. [14]. The method trains a Bayesian network that combines a variety of genomic features such as mRNA coexpression, colocalization, etc. to derive interaction confidence scores for protein pairs. The authors used protein interactions derived from a set of manually curated protein complexes as the set of positive training examples and pairs of proteins localized to different cellular compartments as the set of negative training examples. We refer to this network as the *BAYESIAN network*.

Table 1 summarizes the structural properties of the six networks just described. (Here and throughout the paper we analyze the largest connected component of each protein interaction network.) Table 2 shows the overlap, fraction of interactions in common, between the networks. Given the differences in the experimental techniques used to construct these networks and the fact that the edges in the TAP-MS and BAYESIAN networks correspond to membership in multiprotein complexes, in the Y2H to physical contacts, and in the DIP CORE, LC, and HC networks to a mix of these two things, it is not surprising that the networks differ significantly in terms of density, cliquishness, and other parameters. The biggest outlier is the Y2H network. In fact, for this network, the relation between essentiality and lethality is less prominent as discussed in the next section.

**Table 1:** Structural properties of the tested protein interaction networks.

	Number of nodes	Number of edges	Average degree	Average clustering coefficient
DIP CORE	2,316	5,569	4.81	0.30
LC	3,224	11,291	7.00	0.36
HC	2,752	9,097	6.61	0.37
TAP-MS	1,994	15,819	15.87	0.60
BAYESIAN	4,135	20,984	10.15	0.26
Y2H	400	491	2.45	0.09

**Table 2:** Amount of overlap between tested networks.

DIP CORE	0.58	0.62	0.25	0.61	0.02
0.28	LC	0.53	0.26	0.39	0.01
0.38	0.65	HC	0.47	0.47	0.02
0.09	0.18	0.27	TAP-MS	0.36	0.00
0.16	0.21	0.20	0.27	BAYESIAN	0.02
0.26	0.18	0.31	0.10	0.97	Y2H

Each row of the table corresponds to a single network and shows a fraction of its edges contained in other tested networks. Thus, for example, 58% of the edges in the DIP CORE network are also present in the LC network.

### The Centrality-Lethality Rule Holds in the Six Networks

In their influential paper, Jeong et al. [3] observed that the degree of a node in a yeast protein interaction network correlates with the phenotypic effect of its deletion. More specifically, the authors observed that high-degree nodes are three times more likely to be essential than nodes having few interaction partners. It was further hypothesized that high-degree nodes tend to be essential due to the central role that they play in maintaining the overall connectivity of the network by mediating interactions among other less connected proteins. Consequently, high-degree nodes are also referred to as *hubs*, and the observed phenomenon is known as *the centrality-lethality rule*.

**Figure 1:**
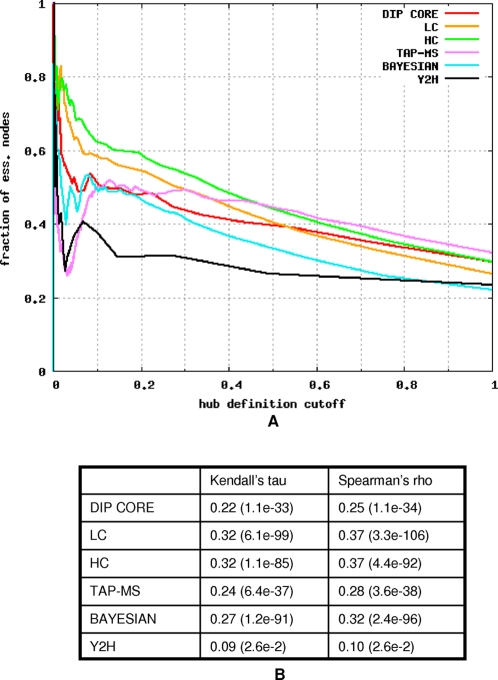
Relationship between degree and essentiality in the tested networks. (A) For each tested network the fraction of essential nodes among nodes with highest degree (hubs) is shown. The horizontal axis shows the fraction of the total network nodes that were designated as hubs. (B) Correlation between degree and essentiality is assessed by Kendall's tau and Spearman's rho rank correlation coefficients.

To confirm the centrality-lethality rule in the tested networks we used the results of a systematic gene deletion screen [25] in which 1,105 yeast genes were found to be essential for growth on rich glucose media. There are numerous ways of exposing positive correlation between degree and essentiality, two of which are used in this paper. First, one can ask whether hubs, nodes with a degree greater than or equal to a certain threshold, are more likely to be essential than an average network node, i.e., whether the fraction of essential proteins among hubs is greater than the network average. To choose an appropriate threshold value we relied on Figure 1A, which shows the enrichment values for nodes with a degree greater than or equal to *k* as a function of *k*. In some networks the steady increase of enrichment values is interrupted for very large values of *k*. Therefore, we chose the threshold value so that approximately 20% of the network nodes are hubs. (For the DIP CORE network the value of *k* is 7, for the LC network it is 10, for the HC network it is 10, for the TAP-MS network it is 24, for the BAYESIAN network it is 12, and for the Y2H network it is 3.) However, we repeated the experiments with hubs defined as 10% (data not shown) and found that our conclusions are robust to the specific choice of the threshold.

From Figure 1A it is clear that the enrichment values increase with *k*. Therefore, one can use a nonparametric measure of association, such as *Kendall's tau* and *Spearman's rho rank correlation coefficients* [26], to assess the correlation between degree and essentiality over all network nodes. As shown in Figure 1B these two measures agree in their estimates of the strength of the correlation; therefore all further evaluations were done with the Kendall's tau rank correlation coefficient. Then, to assess the correlation between other centrality measures and essentiality after correcting for correlation with degree, we used a partial Kendall's tau rank correlation.

It should be noted that in contrast to other networks the Y2H network exhibits only a weak correlation between degree and essentiality. This is in agreement with the study of Batada et al. [4]. They observed a highly significant difference in the average degree of essential and nonessential proteins in the LC network but found that the difference almost disappears when the analysis is restricted to interactions detected by only the yeast-two-hybrid experiments.

### Essential Hubs Are Not More Important in Maintaining the Overall Network Connectivity Than Nonessential Hubs

A network centrality index assigns a centrality value to each node in the network that quantifies its topological prominence. Topological prominence can be defined in a number of ways, and over the years many centrality indices were introduced that emphasize different aspects of network topology [27]. In a local centrality index, the node's centrality value is mainly influenced by the topology of its local neighborhood. A well known example of a local centrality index is degree centrality, where the node's centrality value is equal to the number of its immediate neighbors. Betweenness indices, on the other hand, assign centrality values based on the node's role in maintaining the connectivity between pairs of other nodes in the network. A well-known example of a betweenness centrality index is shortest-path betweenness centrality, where the node's centrality value is proportional to the fraction of shortest paths that pass through it.

**Figure 2:**
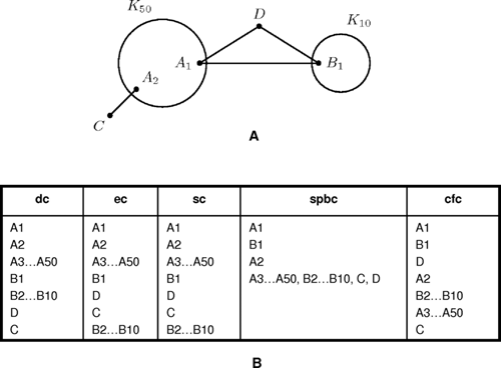
Centrality measures demonstrated on a toy network. Here we demonstrate the difference in the five centrality measures on a toy network. (A) The toy network consists of two cliques: K_50_ with nodes A_1_–A_50_ and K_10_ with nodes B_1_–B_10_. The two cliques are interconnected by an edge (A_1_, B_1_) and through an additional vertex D. Additional node C attaches to the network through A_2_. (B) As the measures assign centrality values based on different network properties they will rank nodes differently. Briefly, the eigenvector centrality measure (EC) will assign high-centrality values to nodes that are close to many other central nodes in the network. The subgraph centrality measure (SC) assigns centrality values to a node based on the number of closed walks that originate at the node. The shortest path betweenness centrality measure (SPBC) assigns the node centrality value based on the fraction of shortest paths that pass through the node averaged over all pairs of nodes in the network. The current-flow betweenness centrality measure (CFC) generalizes the SPBC measure by including additional paths, not just the shortest paths, in the computation. Here, the difference between the measures is exemplified by the rankings that they produce for the toy network nodes.

Even though degree centrality is a local centrality index, in some networks hubs may play an important role in maintaining the overall connectivity of the network. For example, it was demonstrated that in some scale-free networks the removal of hubs affects the ability of other nodes to communicate much more than the removal of random nodes [8]. To clarify the topological role of hubs in the tested networks, we compared degree centrality to two other local indices (eigenvector centrality (EC) [28] and subgraph centrality (SC) [29]) and to two betweenness indices (shortest-path betweenness centrality (SPBC) [30] and current-flow betweenness centrality (CFC) [31]). (See Figure 2 for an illustration, and Materials and Methods for a more detailed description of the centrality measures used in this study.)

**Figure 3:**
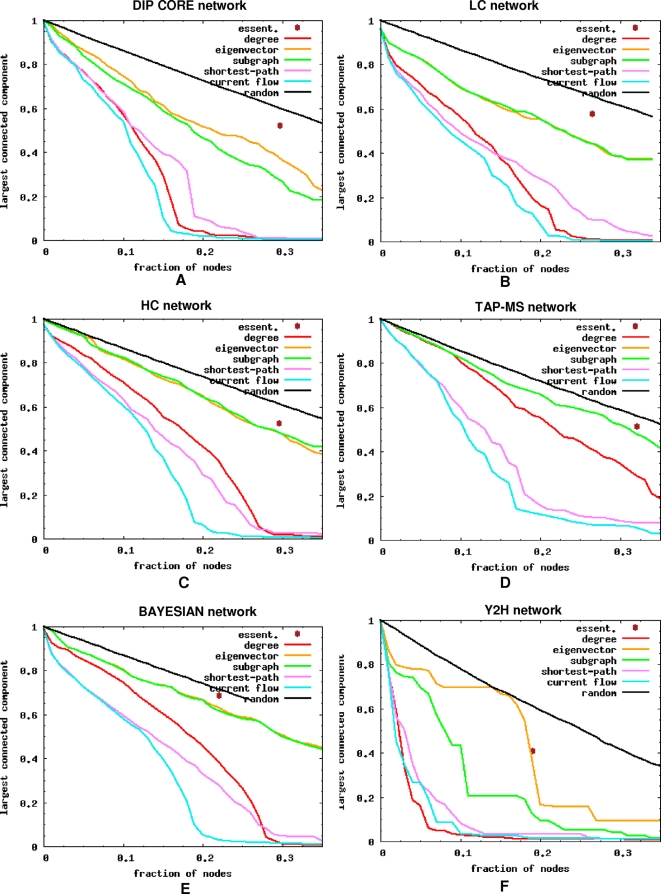
Vulnerability to attack against most central proteins. (A–F) The impact of node removal is quantified by the fraction of nodes in the largest connected component. There is one curve for each centrality measure that shows the fraction of nodes in the largest connected component as a function of the fraction of the most central nodes removed. We also show the impact of node removal in a random order and the size of the largest connected component when all essential proteins are removed.

Since betweenness indices rank nodes based on their role in mediating communication between pairs of other nodes in the network, it is interesting to compare the effectiveness of high-degree nodes and nodes with high betweenness centrality in disconnecting the network. One common way to measure the impact of the nodes' removal on the network connectivity is by monitoring the decrease in the size of the largest connected component. Figure 3A–F shows, for the six protein interaction networks, how the removal of the most central nodes, random nodes, and essential proteins affects the network connectivity. As expected, removing nodes with high local centrality values is much less disruptive than removing those with high betweenness centrality values. Interestingly, degree centrality is as efficient in shattering the network as betweenness in the DIP CORE, LC, and Y2H networks, is as inefficient as the local indices in the TAP-MS network, and is somewhere between the local and betweenness indices in the HC and BAYESIAN networks. The local measures strongly agree in their ranking of network nodes in all networks except the Y2H network. The agreement is the strongest in the TAP-MS network; as a result the curves for the EC and SC measures overlap completely in Figure 3D.

While the removal of a set of nodes may not disconnect various parts of the network, it may impair significantly the “quality of communication” between them. For example, there can be an increase in the length of the shortest path or decrease in the number of alternative paths between pairs of nodes in the network. Therefore, we introduced two additional measures, which we call network integrity measures, to capture various aspects of the effect of the nodes' removal on the ability of other nodes to communicate. (See Materials and Methods for a description of the network integrity measures.) We find that even when these more sensitive measures are used the observations made above about the disruptive power of hubs relative to other most central proteins hold (Table S1).

Next, we examined whether the disruption power of hubs comes mainly from essential hubs. First, we observe that the removal of all essential proteins from the huge connected component is less disruptive than the removal of an equivalent number of the most central nodes according to any index (Figure 3A–F). Moreover, as shown in Table 3, the removal of essential nodes is not more disruptive than the removal of an equivalent number of random nonessential nodes that have the same degree distribution. We conclude that even though in most networks, the DIP CORE, LC, HC, and Y2H networks, the removal of high-degree nodes is disruptive, this disruption is not related to the essentiality of these nodes. On the contrary, essential genes are indistinguishable in that respect from the random nonessential genes with the same degree distribution.

**Table 3:** Impact of the removal of essential proteins as compared to the removal of an equivalent number of random nonessential proteins with the same degree distribution.

	Essential	Random nonessential
DIP CORE	0.519	0.504±0.007
LC	0.578	0.551±0.010
HC	0.521	0.525±0.005
TAP-MS	0.512	0.512±0.011
BAYESIAN	0.685	0.625±0.006
Y2H	0.410	0.397±0.046

The impact of removal of a set of proteins is measured by the fraction of nodes in the largest connected component. For each network the effect of the removal of essential proteins and the removal of an equivalent number of random nonessential proteins with the same degree is shown.

### There Is No Relationship between the Disruptive Power of a Centrality Index and Its Enrichment in Essential Proteins

**Figure 4:**
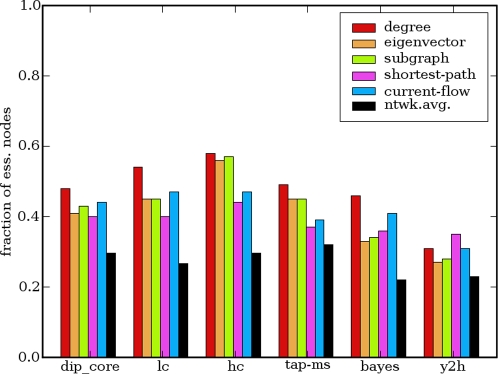
Enrichment of hubs and an equivalent number of most central nodes according to other centrality measures in essential proteins. Fraction of essential proteins among hubs and an equivalent number of most central nodes according to four other centrality measures. The fraction of essential proteins among the nodes of the network is shown as ntwk.avg.

Above we demonstrated that various centrality indices vary considerably in their ability to predict disruption in the overall connectivity of the network. Next we asked whether this difference is reflected in the enrichment levels. Figure 4 shows the fraction of essential proteins among hubs and an equivalent number of most central proteins according to five centrality measures. We observe that the local centrality indices have enrichment levels comparable to those of betweenness indices and in some cases even higher. But most notably, degree centrality fares better than any other centrality index in five networks but is narrowly beaten by shortest-path centrality for the Y2H network. The superiority of degree centrality is even more apparent when Kendall's tau rank correlation coefficient is used to measure correlation between centrality values and essentiality over all network nodes (compare Table 2 to Table 4).

**Table 4:** Correlation between centrality indices and essentiality.

	Eigenvector centrality		Subgraph centrality	
	*τ*_ess_	*τ*_ess.dc_	*τ*_ess_	*τ*_ess.dc_
DIP CORE	0.15 (3.5e-19)	0.064 (8.6e-05)	0.17 (1.2e-24)	0.059 (2.5e-04)
LC	0.23 (7.9e-56)	0.094 (3.6e-11)	0.23 (1.2e-55)	0.093 (4.9e-11)
HC	0.24 (1.8e-54)	0.107 (2.9e-12)	0.24 (7.9e-55)	0.102 (3.4e-11)
TAP-MS	0.12 (8.42e-11)	−0.007 (6.5e-01)	0.12 (8.42e-11)	−0.007 (6.5e-01)
BAYESIAN	0.17 (5.7e-39)	0.046 (1.5e-04)	0.17 (5.1e-41)	0.051 (3.1e-05)
Y2H	0.05 (1.1e-01)	0.027 (2.5e-01)	0.03 (2.0e-01)	−0.024 (7.2e-01)
	Shortest-path betweenness centrality		Current-flow betweenness	
	*τ*_ess_	*τ*_ess.dc_	*τ*_ess_	*τ*_ess.dc_
DIP CORE	0.15 (3.2e-18)	−0.002 (5.5e-01)	0.19 (2.7e-27)	0.012 (2.5e-01)
LC	0.21 (1.4e-46)	0.003 (4.25e-01)	0.26 (3.7e-70)	−0.007 (6.8e-01)
HC	0.20 (1.9e-36)	0.005 (3.7e-01)	0.24 (2.6e-53)	−0.005 (6.2e-01)
TAP-MS	0.12 (3.5e-11)	0.018 (1.8e-01)	0.16 (3.3e-18)	0.017 (1.8e-01)
BAYESIAN	0.18 (2.4e-41)	0.005 (3.43e-01)	0.23 (2.7e-69)	0.018 (8.1e-02)
Y2H	0.10 (1.2e-02)	0.048 (1.4e-01)	0.10 (1.4e-02)	0.041 (1.8e-01)

The correlation of centrality measures with essentiality (*τ*_ess_) is measured by Kendall's tau rank correlation coefficient. The correlation with essentiality, after controlling for correlation with degree centrality, is measured using the partial Kendall's tau rank correlation coefficient (*τ*_ess.dc_). The *p*-values are derived from the Kendall's tau *z*-scores and are shown in parentheses.

As there is considerable correlation between degree centrality and other centrality indices, we used Kendall's tau partial rank correlation coefficient to see whether any of the indices is correlated with essentiality beyond its correlation with degree centrality index. We found that, controlling for the correlation with degree, the correlation with essentiality is reduced to statistically insignificant values for betweenness centrality indices and is greatly reduced for local indices (Table 4).

The above observations indicate that the main topological determinant of essentiality is the node's local neighborhood rather than its role in maintaining the overall connectivity of the network. In particular, even though removing the nodes with high betweenness centrality indices is much more effective in shattering some of our protein interaction networks, their correlation with essentiality is reduced to statistically insignificant levels by subtracting their correlation with degree centrality.

### We Reject the Essential Protein Interaction Model

Recently He and colleagues [9] proposed an explanation for the centrality-lethality rule in terms of essential protein interactions: a protein is essential either due to its involvement in one or more essential protein interactions or due to other factors. The authors argue that the determination of protein essentiality in the protein interaction network can be captured by a simple random process: (i) distribute essential protein interactions along the edges of the network uniformly at random with probability *α*; (ii) distribute essential proteins among the nodes of the network uniformly at random with probability *β*. Thus, according to the model, the probability (*P*_E_) of a protein with *k* neighbors being essential is *P*_E_ = 1−(1−*α*)*^k^*(1−*β*), and the natural logarithm of the fraction of nonessential proteins among proteins of degree *k* has a linear dependency on *k*: log(1−*P*_E_) = log(1−*α*)*k*+log(1−*β*).

We note that from the assumptions of the essential protein interaction model it follows that if two proteins do not interact then the essentiality of one protein in such a pair does not depend on the essentiality of the other protein. Furthermore, this independence should also be observed when proteins share interaction neighbors. To test whether this holds in real data, we computed the number of nonadjacent protein pairs, with three or more neighbors (one or more neighbors in the Y2H network), that are either both essential or both nonessential in the tested networks and compared these numbers to the expected number of such pairs under the model. (The model parameters were estimated using three different strategies as described in the Materials and Methods. In their paper, He et al. point out that their model may not work in networks where the edges represent membership in the same protein complex. Thus, we excluded the TAP-MS and BAYESIAN networks from the analysis.) As shown in Table 5, the model does not capture the correlation in essentiality observed in the tested networks; i.e., there is a statistically significant difference between the number of such pairs observed in real data and the number expected under the model. Consequently, the essential interaction model is rejected with high confidence.

**Table 5:** Difference between the observed and expected number of pairs where both proteins are either essential or nonessential.

	Total number of pairs	Number of pairs of the same type	Expected number of pairs of the same type		
			Simulation	Line fitting	Weighted line fitting
DIP CORE	1,849	1,135	945 (3.6e-10)	928 (8.6e-12)	938 (8.0e-11)
LC	10,777	6,143	5,691 (6.6e-10)	5.556 (1.1e-15)	5.589 (3.9e-14)
HC	5,907	3,516	3,213 (2.0e-08)	2,997 (2.2e-16)	2,994 (2.2e-16)
Y2H	3,254	2,167	1,976 (9.6e-07)	2,025 (2.6e-04)	2,052 (3.3e-03)

The total number of pairs refers to the number of nonadjacent protein pairs with three or more common neighbors in the network. (Due to the sparsity of the Y2H network, the statistics are calculated for nonadjacent pairs having one or more neighbors in common.) The nodes in the pair are of “the same type” if they are both essential or both nonessential.

### We Propose an Alternative Explanation for the Centrality-Lethality Rule

In the previous section we showed that proteins that share neighbors are more likely to have the same essentiality (be both essential or both nonessential) than expected under the essential PPI model. Moreover, it was observed in another study that essential proteins are not distributed uniformly among in the set of automatically derived multiprotein complexes [17]. This suggests that densely connected subnetworks are polarized toward being either highly enriched or significantly depleted of essential proteins. Furthermore, it is well known that densely connected subnetworks are enriched in proteins that share biological function. Therefore, one should expect that protein interaction networks contain densely connected functional modules that are highly enriched in essential proteins. Some large multiprotein complexes, for example, those involved in transcription regulation, are known to be highly enriched in essential proteins, but how general is this phenomenon and can it account for the centrality-lethality rule?

To investigate the above question, we introduce a notion of *Essential Complex Biological Modules*, which are groups of proteins with shared biological function that extensively interact with each other and are enriched in essential proteins. First, we describe an automatic method for the extraction of ECOBIMs from a protein interaction network. Next, we argue that the membership in ECOBIMs accounts to large extent for the centrality-lethality rule in the tested networks. Finally, we address statistical issues related to our selection procedure by applying suitable randomization protocols.

**Figure 5:**
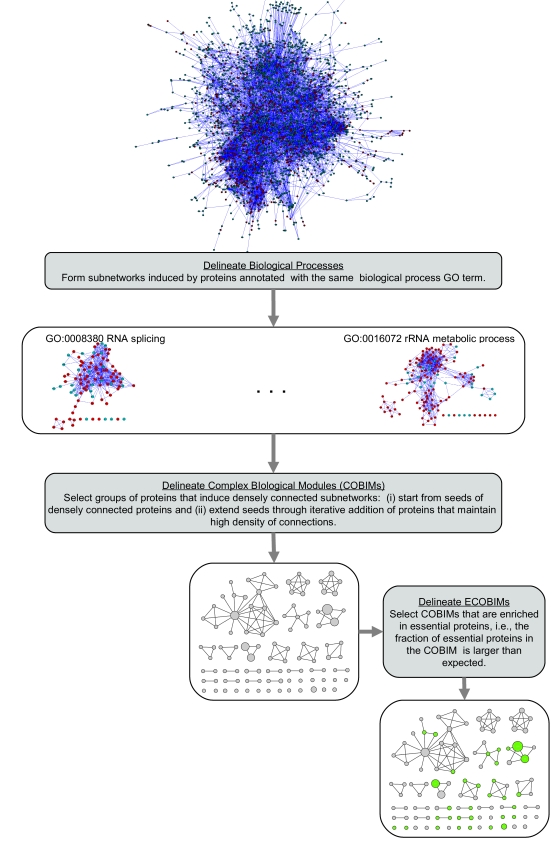
The automatic method for extraction of ECOBIMs. Here we demonstrate the major steps of the method on the HC network. The input to the method is a protein interaction network, GO annotation, and the set of essential nodes, which are shown in red. The method considers subnetworks induced by proteins annotated with the same GO biological process term, one subnetwork at a time, to identify densely connected regions or COBIMs. The COBIMs are shown by a COBIM intersection graph, where nodes correspond to COBIMs (the size of the node is proportional to the number of genes in the corresponding COBIM) and there is an edge between a pair of COBIMs if they have at least two proteins in common. The COBIMs that are enriched in essential proteins are selected as ECOBIMs, shown in green.

We developed an automatic method for extraction of ECOBIMs from a protein interaction network. In this work proteins are deemed to share biological function if they are annotated with the same GO biological process term from a set of 192 terms that were selected by a group of experts to represent relevant aspects of molecular biology [32]. Therefore, our method is applied to subnetworks induced by proteins annotated with the same GO biological process term, one subnetwork at a time. The high-level idea behind the method is to first identify groups of densely connected proteins, which we call *Complex Biological Modules* (or COBIMs), and then identify a subset of COBIMs as ECOBIMs based on the distribution of essential proteins among the COBIM nodes. More specifically, our heuristic selects a subset of COBIMs that are enriched in essential proteins. (The method is schematically shown in Figure 5 and is described in detail in the Materials and Methods section. Figure S1 shows the fraction of nodes that are members of *r* or more COBIMs for various values of *r*.)

**Figure 6:**
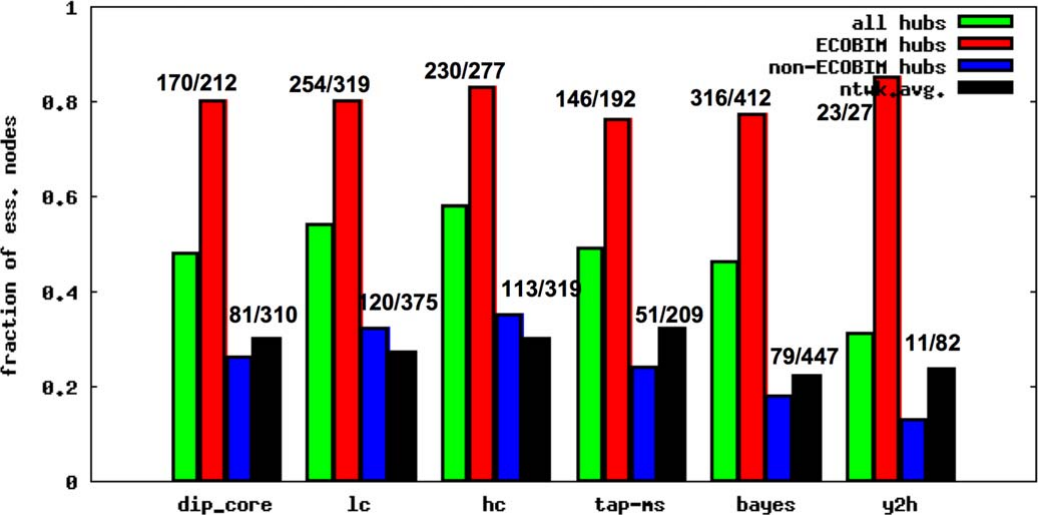
Enrichment of ECOBIM and non-ECOBIM hubs in essential proteins. Fraction of essential proteins among various types of hubs: all hubs, hubs that are members of ECOBIMs (ECOBIM hubs), and hubs that are not members of ECOBIMs (non-ECOBIM hubs). The fraction of essential proteins among all proteins in the network is also shown (ntwk.avg.). The numbers above the bars show the number of essential hubs out of the total number of hubs of this type for ECOBIM and non-ECOBIM hubs.

To examine to what extent the membership in ECOBIMs accounts for the centrality-lethality rule we partitioned hubs into two groups, those that are members of one or more ECOBIMs (ECOBIM hubs) and those that are not (non-ECOBIM hubs), and compared their enrichment values. As shown in Figure 6 ECOBIM hubs are highly enriched in essential proteins, whereas non-ECOBIM hubs are depleted in essential proteins as compared to the network average enrichment values. But most importantly, as discussed in the next paragraph, the difference in the fraction of essential proteins among ECOBIM hubs and non-ECOBIM hubs is not a result of our greedy ECOBIM selection procedure or particular degree sequence of essential proteins in the network. We next asked whether there is a correlation between degree and lethality for network nodes that are not members of the ECOBIMs. As shown in Table 6 the correlation between essentiality and degree for non-ECOBIM nodes is much less than that for all network nodes.

**Table 6:** Membership in ECOBIMs and the centrality-lethality rule.

	Enrichment of ECOBIM hubs			Enrichment of non-ECOBIM hubs			Corr. degree vs. essentiality for non-ECOBIM hubs		
	Obs.	Rand.	*p*-value	Obs.	Rand.	*p*-value	Obs.	Rand.	*p*-value
DIP CORE	0.80	0.67	1.98e-03	0.26	0.43	<1.00e-05	0.08	0.18	<1.00e-05
LC	0.80	0.69	1.88e-03	0.32	0.48	<1.00e-05	0.17	0.27	<1.00e-05
HC	0.83	0.70	4.00e-05	0.35	0.51	<1.00e-05	0.17	0.27	<1.00e-05
TAP-MS	0.76	0.62	1.00e-05	0.24	0.40	<1.00e-05	0.12	0.20	<1.00e-05
BAYESIAN	0.77	0.65	<1.00e-05	0.18	0.36	<1.00e-05	0.09	0.20	<1.00e-05
Y2H	0.85	0.66	5.81e-02	0.13	0.25	2.00e-05	−0.04	0.05	2.00e-04

For every quantity three values are shown: the value under the true assignment of essential proteins (Obs.), the mean value under the randomized assignment of essential proteins (Rand.), and the fraction of the randomized assignments that resulted in values stronger (either smaller or larger depending on the context) than those obtained with the true assignment of essential proteins (*p*-value).

One may ask to what extent the difference in the behavior of ECOBIM hubs and non-ECOBIM hubs is due to the particular selection procedure that we employ to identify the putative ECOBIMs. More specifically, there are two concerns that need to be addressed. First, our method is guided by the enrichment in essential proteins when selecting ECOBIMs from COBIMs. Therefore, it is expected that the fraction of essential proteins among ECOBIM hubs should be higher than that among non-ECOBIM hubs. Second, our method considers only annotated yeast genes. Therefore, one might argue that the difference in behavior is due to the fact that ECOBIM hubs are necessarily annotated while non-ECOBIM hubs may include both annotated and unannotated genes.

To address the first concern we performed a control experiment where essential proteins were assigned to a random set of nodes having the same degree distribution as the true set of essential proteins in the network. (A total of 100,000 random assignments were performed, which resulted in 100,000 sets of ECOBIMs.) To address the second concern, we restricted the random assignment to annotated genes only. As shown in Table 6, the ECOBIMs resulting from the true assignment of essential proteins have dramatically different properties than these resulting from the random assignment of essential proteins. In particular, the fraction of essential proteins among non-ECOBIM hubs under the true assignment of essential proteins is significantly lower than that under the randomized assignment of essential proteins, even though the same selection procedure is used in both cases. Therefore, we conclude that the observed difference is the result of the particular distribution of essential proteins among the nodes of the network and not an artifact of our selection procedure. The same holds for the reduction in correlation between degree and essentiality for non-ECOBIM nodes.

### We Identify Properties of the ECOBIMs

The identified ECOBIMs mostly correspond to large essential multiprotein complexes such as the anaphase promoting complex (APC) and the DAM1 protein complex but not exclusively complexes. For example, one of the largest ECOBIMs identified in the LC network contains multiprotein complexes involved in the process of RNA polymerase 2 transcription [33], such as RNA polymerase 2, general transcription factors, the mediator complex, etc. The ECOBIMs with at least 20 members are shown in Table 7; all ECOBIMs and their member proteins are given in Table S2.

**Table 7:** Largest ECOBIMs extracted from the tested networks.

**The DIP CORE network**			
GO:0006508 proteolysis	27	35	0.77
GO:0042254 ribosome biogenesis and assembly	27	32	0.84
GO:0016192 vesicle mediated transport	21	30	0.70
GO:0016071 mRNA metabolic process	18	28	0.64
GO:0015931 nucleobase, nucleoside, nucleotide and nucleic acid transport GO:0051236 establishment of RNA localization	15	24	0.62
GO:0016072 rRNA metabolic process	18	21	0.86
GO:0008380 RNA splicing	16	21	0.76
**The LC network**			
GO:0042254 ribosome biogenesis and assembly	88	107	0.82
GO:0016071 mRNA metabolic process	37	58	0.64
GO:0008380 RNA splicing	35	52	0.67
GO:0015931 nucleobase, nucleoside, nucleotide and nucleic acid transport GO:0051236 establishment of RNA localization	16	26	0.62
GO:0006508 proteolysis	17	24	0.71
**The HC network**			
GO:0042254 ribosome biogenesis and assembly	84	100	0.84
GO:0016071 mRNA metabolic process	49	71	0.69
GO:0016072 rRNA metabolic process	63	71	0.89
GO:0008380 RNA splicing	46	63	0.73
GO:0006508 proteolysis	28	35	0.80
**The TAP-MS network**			
GO:0042254 ribosome biogenesis and assembly	90	120	0.75
GO:0016071 mRNA metabolic process	46	66	0.70
GO:0008380 RNA splicing	45	62	0.73
GO:0016072 rRNA metabolic process	37	41	0.90
GO:0016072 rRNA metabolic process	30	32	0.94
GO:0006508 proteolysis	17	22	0.77
**The BAYESIAN network**			
GO:0042254 ribosome biogenesis and assembly	119	152	0.78
GO:0016072 rRNA metabolic process	93	106	0.88
GO:0008380 RNA splicing GO:0016071 mRNA metabolic process	40	50	0.80
GO:0006366 transcription from RNA polymerase II promoter	23	42	0.55
GO:0006508 proteolysis	28	37	0.76
GO:0006913 nucleocytoplasmic transport	17	31	0.55
GO:0006412 translation	18	27	0.67
GO:0051169 nuclear transport	15	27	0.55
GO:0045184 establishment of protein localization	15	27	0.55
**The Y2H network**			
GO:0007010 cytoskeleton organization and biogenesis	9	11	0.82
GO:0006366 transcription from RNA polymerase II promoter	7	11	0.64
GO:0045184 establishment of protein localization	6	10	0.60
GO:0006913 nucleocytoplasmic transport GO:0051169 nuclear transport	6	10	0.60

For every tested protein interaction network we list the ECOBIMs with at least 20 members; for the Y2H network, the ECOBIMs with at least 10 members are listed. For each ECOBIM the following information is shown: the corresponding GO biological process term, number of essential genes, number of genes, and fraction of essential genes. For a list of all ECOBIMs and their member genes see Table S2.

Moreover, the ECOBIMs are remarkably different than non-ECOBIM COBIMs. As shown in Table 8, the distribution of essential proteins among the COBIM nodes is highly uneven. In particular, the observed difference between fractions of essential proteins among the ECOBIM nodes and among non-ECOBIM COBIM nodes can not be accounted for neither by degrees of essential COBIM nodes nor by the particular ECOBIM selection procedure. The last claim is validated by performing 100,000 randomized assignments of essential proteins that preserve degrees and the number of essential COBIM nodes, selecting the ECOBIMs and computing the corresponding fractions. As shown in Table 8, the values obtained under the true assignment of essential proteins are significantly different from those obtained under the randomized assignment of essential proteins.

**Table 8:** ECOBIMs contain a large fraction of essential COBIM proteins.

	Enrich. ECOBIM proteins			Enrich. non-ECOBIM COBIM proteins		
	Obs.	Rand.	*p*-value	Obs.	Rand.	*p*-value
DIP CORE	0.77	0.65	<1.0e-05	0.06	0.21	<1.0e-05
LC	0.77	0.65	1.00e-05	0.10	0.17	1.56e-03
HC	0.81	0.68	<1.00e-05	0.12	0.18	2.31e-02
TAP-MS	0.74	0.64	<1.00e-05	0.09	0.17	1.87e-03
BAYESIAN	0.76	0.65	<1.00e-05	0.08	0.18	<1.00e-05
Y2H	0.79	0.63	9.93e-03	0.06	0.17	3.00e-05

For each network the enrichment in essential proteins of ECOBIM nodes and enrichment of COBIM nodes that are not members of one or more ECOBIMs is shown. For each group three values are listed: the fraction under the true assignment of essential proteins (Obs.), the mean fraction under the randomized assignment of essential proteins (Rand.), and *p*-value of the difference.

**Figure 7:**
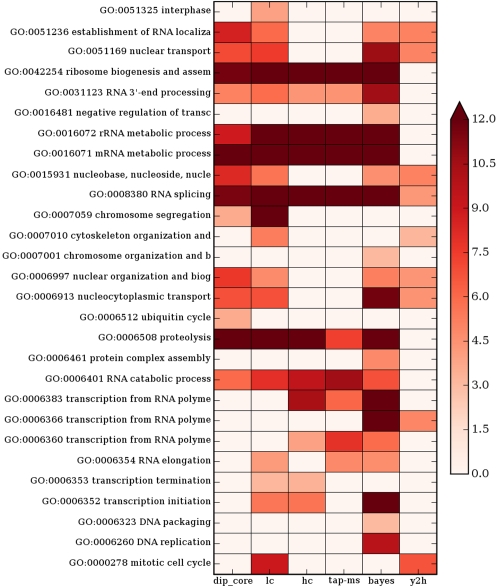
GO terms that are overrepresented among ECOBIM nodes. For every network the GO terms that are overrepresented among ECOBIM nodes are shown. The overrepresentation of a GO term is quantified by the natural logarithm of a *p*-value, where the *p*-value is the probability that at least this number of ECOBIM genes would belong to the GO term had the ECOBIM genes been selected uniformly at random from the network genes.

So far, we demonstrated that the high correlation between degree and essentially can be predominantly attributed to the ECOBIMs. In addition, it is well known that certain functions that are essential to the cell, for example, transcription regulation or cell-cycle regulation, rely on large multiprotein complexes. Indeed, many of the GO terms that are overrepresented among ECOBIM nodes are of this type, as seen in Figure 7. Do ECOBIMs play a distinguished role in those essential processes, or are they merely a byproduct of the above-mentioned observation? In particular, is the difference in the enrichment in essential proteins exclusively due to the fact that some essential GO processes contain ECOBIMs while others do not?

To elucidate the role of the ECOBIMs we examined all GO processes that contain at least one ECOBIM. Table 9 shows the results for the DIP core network sorted by the percentage of essential proteins in a given GO process. (The data for the other networks are given in Table S3.) Observe that the enrichment of ECOBIMs in essential genes is typically much higher than the average enrichment in the corresponding GO. Thus, the ECOBIMs are not merely representatives of the average structure of the corresponding GO subnetwork. The uneven distribution of essential proteins is also observed even when the corresponding GO process is extremely enriched in essential proteins such as rRNA metabolic process (GO:0016072) or transcription initiation (GO:0006352). The percentage of essential proteins among network nodes annotated to either one of these two processes is more than 80%, and all COBIMs are selected as ECOBIMs. The process with the next highest percentage of essential proteins, transcription from RNA polymerase III promoter (GO:0006383), contains both types of COBIMs. Interestingly, for this process, all ECOBIM nodes are essential, but none of the remaining COBIM nodes is. In fact, if a GO process contains both ECOBIM and non-ECOBIM COBIMS, then such polarization is frequent albeit rarely that extreme (Table 9). Removal of any protein from a Complex Biological Module is expected to perturb or even disable the whole module. Thus, within a large spectrum of essential GO processes, a cell can tolerate large perturbations of some modules but very little perturbations of ECOBIMs.

**Table 9:** Enrichment of ECOBIM and non-ECOBIM COBIM nodes for GO subnetworks in the DIP CORE network.

GO term	Subnetwork nodes	ECOBIM nodes	Non-ECOBIM COBIM nodes
GO:0016072 rRNA metabolic process	0.83	0.91	n/a
GO:0006352 transcription initiation	0.82	1.00	n/a
GO:0006383 transcription from RNA polymerase III pro	0.77	1.00	0.00
GO:0042254 ribosome biogenesis and assembly	0.72	0.87	n/a
GO:0008380 RNA splicing	0.71	0.79	0.50
GO:0006839 mitochondrial transport	0.64	0.80	n/a
GO:0006360 transcription from RNA polymerase I pro	0.64	0.80	0.00
GO:0016071 mRNA metabolic process	0.63	0.75	0.40
GO:0006260 DNA replication	0.61	0.93	n/a
GO:0031123 RNA 3′-end processing	0.59	0.93	0.29
GO:0006399 tRNA metabolic process	0.50	1.00	0.00
GO:0007059 chromosome segregation	0.49	0.76	n/a
GO:0006944 membrane fusion	0.48	0.75	0.22
GO:0006508 proteolysis	0.46	0.77	n/a
GO:0051169 nuclear transport	0.44	0.80	0.47
GO:0006997 nuclear organization and biogenesis	0.43	1.00	0.33
GO:0000278 mitotic cell cycle	0.43	0.81	0.19
GO:0015931 nucleobase, nucleoside, nucleotide and n	0.42	0.63	n/a
GO:0006913 nucleocytoplasmic transport	0.42	0.80	0.41
GO:0051236 establishment of RNA localization	0.42	0.63	n/a
GO:0006366 transcription from RNA polymerase II pro	0.40	0.75	0.29
GO:0007010 cytoskeleton organization and biogenesis	0.40	0.78	0.00
GO:0048308 organelle inheritance	0.39	0.86	n/a
GO:0006401 RNA catabolic process	0.38	0.83	0.41
GO:0006461 protein complex assembly	0.38	1.00	n/a
GO:0045184 establishment of protein localization	0.37	0.89	0.38
GO:0009100 glycoprotein metabolic process	0.37	0.63	n/a
GO:0006412 translation	0.36	0.85	0.00
GO:0007005 mitochondrion organization and biogenes	0.35	0.91	n/a
GO:0006512 ubiquitin cycle	0.34	0.82	n/a
GO:0051325 interphase	0.33	0.83	0.00
GO:0016192 vesicle-mediated transport	0.31	0.71	0.18
GO:0000074 regulation of progression through cell cycl	0.31	0.73	0.18
GO:0000279 M phase	0.30	0.80	0.17
GO:0006974 response to DNA damage stimulus	0.28	0.67	0.11
GO:0006323 DNA packaging	0.26	1.00	0.16
GO:0006417 regulation of translation	0.26	0.80	n/a
GO:0016481 negative regulation of transcription	0.25	1.00	0.13
GO:0007001 chromosome organization and biogenesi	0.22	0.79	0.16
GO:0016458 gene silencing	0.22	1.00	0.00
GO:0040029 regulation of gene expression, epigenet	0.21	1.00	0.00
GO:0007047 cell wall organization and biogenesis	0.17	0.75	n/a

For each GO subnetwork that contributed at least one ECOBIM, the fractions of essential proteins among the subnetwork nodes, subnetwork ECOBIM nodes, and subnetwork non-ECOBIM COBIM nodes are shown.

This last observation can also explain the poor correlation between degree and essentiality in Y2H networks, as it indicates that ECOBIMs are likely to contain large, stable multiprotein modules, typically multiprotein complexes. However, interactions recovered by the Y2H technique correspond to physical contacts and as such do not encompass all members of a complex. Moreover, due to its binary nature, the Y2H technique may completely miss interactions in complexes that require cooperative binding [34].

## Discussion

The enrichment of high-degree nodes in essential proteins, known as the centrality-lethality rule, suggests that the topological prominence of a protein in a protein interaction network may be a good predictor of its biological importance. There exist numerous measures of topological prominence, called network centrality indices; local centrality indices assign centrality values based on the topology of the node's local neighborhood, whereas betweenness centrality indices assign centrality values based on the node's role in maintaining the connectivity between pairs of other nodes in the network. Even though by definition degree centrality is a local measure, depending on the structure of the network, hubs may play an important role in maintaining the overall connectivity of the network. In this paper we sought to identify the main topological determinant of essentiality and to give a biological explanation for the connection between the network topology and essentiality.

To address this question we performed a rigorous analysis of six protein interaction networks for *Saccharomyces cerevisiae* compiled from diverse sources of interaction evidence. To clarify the topological roles of essential proteins in general and essential hubs in particular, we compared degree centrality to other local and betweenness centrality indices. We found that while in some networks high-degree nodes are as important in maintaining the overall network connectivity as nodes having high betweenness centrality values, this property is not due to essential proteins. On the contrary, essential proteins are indistinguishable in that respect from nonessential proteins having the same degree distribution. We also found that degree centrality is a better predictor of essentiality than any other measure tested and that correlation of betweenness indices with essentiality is entirely due to their correlation with degree centrality. Thus, we conclude that the topological determinant of essentiality is the node's local neighborhood rather than its role in maintaining the overall connectivity of the network.

Next we examined whether the essential interactions model, recently proposed to explain the centrality-lethality rule, is valid in the tested networks. We found that the model's central assumption that the majority of proteins are essential due to their involvement in one or more essential protein interactions, which are distributed uniformly at random along the edges of the network, violates basic clustering patterns of essential proteins in the networks that we examined. The uniform distribution of essential protein interactions implies that, as long as two proteins do not interact, the essentiality of one protein in the pair is independent of the essentiality of the other protein. However, in real protein interaction networks the essentiality of pairs of proteins that share many neighbors is correlated, and the number of nonadjacent protein pairs that share three or more neighbors and are either both essential or both nonessential significantly deviates from the expected number of such pairs under the model. Consequently, we rejected the essential interactions explanation with high confidence. We stress that we do not reject the existence of essential protein interactions but rather the assumption that these interactions are evenly distributed along the edges of the network and explain the degree distribution of essential proteins.

The above observations led us to propose an alternative explanation for the centrality-lethality rule. Our explanation builds on a growing body of evidence that gene knock-out phenotypes for genes whose gene products are members of the same multiprotein complex are correlated [16],[17]. In particular, Hart et al. demonstrated that essential proteins are not distributed evenly among the set of automatically identified multiprotein complexes; rather there are “surprisingly” many complexes where the majority of members are essential and “surprisingly” many complexes where the majority of members are not essential [17]. Here we hypothesized and then computationally confirmed that the same phenomenon holds for potentially larger groups of densely connected and functionally related proteins that we called Complex Biological Modules and abbreviated as COBIMs. But more importantly, we were able to demonstrate that membership in ECOBIMs, those COBIMs that are enriched in essential proteins, provides a good explanation for the correlation between degree and essentiality in the protein interaction networks considered in this study. In particular, we showed that non-ECOBIM hubs are depleted in essential proteins and for non-ECOBIM proteins the correlation between degree and essentiality is greatly reduced. Moreover, by applying suitable randomization protocols we showed that the different characteristics of ECOBIM and non-ECOBIM hubs (or in general ECOBIM and non-ECOBIM proteins) are not a mere consequence of their degrees or the particular computational method that we adopted for selecting the ECOBIMs.

In the past, several attempts were made to classify high-degree nodes using additional biological data to obtain a deeper insight into biological and physiological properties that hubs were reported to possess. Here we discuss how our findings fit the results reported in two such studies [35],[36]. Han et al. utilized mRNA expression data to classify hubs into *party* and *date hubs*, where the party hubs show a significant agreement in the mRNA expression levels, or are coexpressed, with their interacting partners, whereas the date hubs are not coexpressed with their neighbors [35]. The removal of the date hubs was observed to shatter the network much more efficiently than the removal of party hubs. On the basis of this and other observations made in the paper, the date hubs were proposed to “…participate in a wide range of integrated connections required for the global organization of biological modules in the whole proteome network…” However, the fraction of essential proteins among the party hubs was even slightly higher than that among the date hubs. This is consistent with one of the conclusions made in this paper, namely, essentiality is not a byproduct of the node's ability to maintain the overall connectivity of the network. Furthermore, it has been proposed that “party hubs represent integral elements within distinct modules” and “tend to function at a lower level of the organization of the proteome” [35]. Such a description is consistent with the properties COBIM hubs where COBIMs hubs are explicitly defined as hubs that are members of highly connected modules. Similarly to the party hubs, the average enrichment of COBIM hubs in essential proteins is slightly higher than that of non-COBIM hubs (data not shown). We also demonstrated that essential proteins clearly cluster within ECOBIMs rather than being uniformly distributed over all COBIMs.

In the second study Kim et al. utilized structural data to classify hubs into *singlish-interface* and *multiinterface hubs*, where singlish-interface hubs would interact with their partners through one or two distinct interfaces, whereas the multiinterface hubs would interact with their partners through three or more distinct interfaces [36]. In this case, however, the classification produced significantly different enrichment levels, with a multiinterface hub being twice as likely to be essential as a singlish-interface hub or an average network node. The authors suggested that multiinterface hubs most likely correspond to members of large and stable multiprotein complexes. Consequently, this would imply that stable multiprotein complexes are enriched in essential proteins. This view is consistent with the results of this paper with additional caveats as discussed below.

It is well known that certain biological functions essential for the cell depend on large multiprotein complexes. (Consider, for example, RNA Polymerase II transcription machinery [33] or ribosome biogenesis and assembly [37].) Indeed, many ECOBIMs indentified by our approach are associated with such processes. However, even within such essential processes, ECOBIMs distinguish themselves as being more enriched in essential proteins than the remaining proteins within the same process. The enrichment in essential proteins of non-ECOBIM COBIMs is usually at the same level and frequently significantly lower than the average enrichment within the corresponding GO process. Thus, within a large spectrum of essential GO processes, a cell can tolerate large perturbations of non-ECOBIM modules but very little perturbation of ECOBIMs. Some COBIMs do not contain any essential proteins. In such a case, the whole module can be nonessential, and the fact that a cell can tolerate the removal of any of member of such a COBIM does not exclude the possibility that this COBIM corresponds to a stable complex.

## Materials and Methods

### Network Centrality Indices

In this work we compare the degree centrality measure to two other local measures (eigenvector centrality (EC) [28] and subgraph centrality (SC) [29]) and to two betweenness measures (shortest-path betweenness centrality (SPBC) [30] and current-flow betweenness centrality (CFC) [31]).

The computation of the eigenvector centrality values can be cast as an iterative process: (i) start with an initial vector of centrality scores 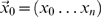; (ii) in iteration *k*+1 update the centrality score of a node *i* using the scores of its neighbors from the previous iteration 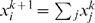 and then normalize the scores 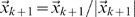. It can be shown that this process converges to the eigenvector that corresponds to the largest eigenvalue of the adjacency matrix of the network.

The subgraph centrality value of a node is equal to the number of closed walks that start and terminate at the node. As there is an infinite number of such walks, to obtain finite index values the number of closed walks of length *k* is weighted by 1/*k*!. Therefore, short walks dominate the subgraph centrality values.

For the shortest-path betweenness index, the node's centrality value is equal to the average fraction of shortest paths that pass through the node.

The current-flow centrality measure extends the shortest-path centrality measure by taking into account other paths in addition to shortest paths. This is achieved through a current-flow paradigm where the network is viewed as a resistor network with each edge having a unit capacity. For every pair of nodes *s* and *t*, one unit of current is shipped from *s* to *t*, and the centrality of a node is set to the average amount of current that passes through that node.

We demonstrate the difference between the five centrality measures on a toy network in Figure 1A. In this network two cliques K_50_ and K_10_ are interconnected by an edge (A_1_, B_1_) and through a node D. The nodes of K_50_ are labeled A_1_…A_50_, and the nodes of K_10_ are labeled B_1_…B_10_. An additional node C attaches to K_50_ through A_2_. Figure 1B shows the ranking of network nodes based on the centrality values assigned by the five centrality measures.

### Network Integrity Measures

We introduced two measures, which we call network integrity measures, to capture various effects of node removal on the ability of other nodes to communicate. An integrity measure maps a set of nodes, *S*, to a value between 0 and 1, with the value of 0 being assigned when the removal of *S* completely disrupts the communication and the value of 1 being assigned when it causes no disruption. Our first measure, shortest-path integrity, quantifies the increase in the length of the shortest path due to the removal of *S* and is given by 
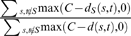
, where *d*(*s*,*t*) is the length of the shortest path between *s* and *t* in the original network, *d_s_*(*s*,*t*) is the length of the shortest path between *s* and *t* after the removal of *S*, and *C* is a constant. In this work we chose the value of *C* to be twice the diameter of the original network. Our second measure, edge-disjoint paths integrity, quantifies the decrease in the number of edge-disjoint paths and is given by 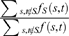, where *f_s_*(*s*,*t*) is the number of edge-disjoint paths between *s* and *t* in the modified network and *f*(*s*,*t*) is this value in the original network.

### Estimating the Parameters of the Essential Protein Interactions Model

To evaluate the model on the tested networks we used three strategies to estimate the model's parameters: a network simulation procedure, line fitting to points (log(1−*P*_E_), *k*) for *k*≤*k*_0_, and weighted line fitting to points (log(1−*P*_E_), *k*) for all values of *k*. (In weighted line fitting, the contribution of (log(1−*P*_E_), *k*) to the error function is weighted by the fraction of nodes having degree *k*.) The first two strategies are described by He et al. [9]. They deem the agreement of parameter values estimated using the network simulation and line fitting strategies to be one of the strongest indications for the validity of the model. But in the tested networks the parameter values estimated using different strategies, as shown in Table S4, vary considerably.

### The Method for Automatic Identification of ECOBIMs

Our method for automatic extraction of putative ECOBIMs is applied to subnetworks induced by proteins annotated with the same biological process GO term. In this work we used a set of 192 biological process terms, which were selected by a group of experts to represent relevant aspects of molecular biology. Thus, the method was applied to 192 subnetworks, one subnetwork at a time.

From each GO subnetwork the method extracts groups of densely connected proteins. An ideal dense network is a *clique*, a complete network where every pair of nodes is adjacent. Over the years various generalizations of the clique concept were proposed in the literature to model a wider set of dense networks. Here we adopt one such generalization based on *k-connectivity*. We say that a pair of nodes is *k-connected* if there are *k* node-disjoint paths in the network between them. We say that a network is *k*-connected if every pair of nodes is *k*-connected. For example, a (*k*+1)-clique, a clique with (*k*+1) nodes, is *k*-connected. In fact, it is the smallest *k*-connected graph.

Our method utilizes the following approach to find regions of GO subnetworks that are *k*-connected: start with a seed that is a (*k*+1)-clique and iteratively extend the seed through addition of proteins that have at least *k* neighbors already in the seed. In addition to being *k*-connected our COBIMs satisfy the following property: nodes can be removed from a COBIM one by one such that the network induced by the remaining nodes is still *k*-connected. We note that not every *k*-connected network has this property. Consider, for example, a cycle. The cycle is 2-connected. However, removal of any node results in a path which is 1-connected. The value of parameter *k* was chosen so that the fraction of COBIM nodes is about 25% of the number of nodes in the network. As shown in Table S5 this results in the following values of *k* for the tested networks: for the DIP CORE network *k* = 3, for the LC and HC networks *k* = 4, for the TAP-MS network *k* = 11, for the BAYESIAN network *k* = 4, and for the Y2H network *k* = 1. We also sampled values of *k* in the neighborhood of selected values and found that the results reported in this paper are however robust with respect to the selected value of *k*. We note that approaches similar to ours have been previously used by Palla et al [38] and Chesler et al. [39].

Once the COBIMs are computed, the method selects a subset of COBIMs based on the distribution of essential proteins among the COBIM nodes. Namely, the heuristic selects all COBIMs with a fraction of essential proteins that is significantly higher than what would be expected from a uniform distribution of essential genes among the COBIM nodes. More specifically, a COBIM with *n* nodes and *m* essential nodes is selected iff:
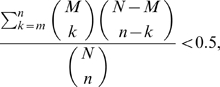
where *N* is the total number of COBIM nodes and *M* is the number of essential COBIM nodes.

## Supporting Information

Figure S1 Membership in COBIMs. The amount of overlap among COBIMs is quantified by showing the fraction of nodes that are members of several COBIMs.
(0.05 MB DOC)

Table S1 Using network integrity measures to evaluate the effect of the removal of hubs and equivalent number of the most central nodes according to other centrality measures
(0.04 MB DOC)

Table S2 ECOBIMs and their member genes. For every tested protein interaction network we list the automatically identified ECOBIMs. For each ECOBIM the following information is shown: the corresponding GO biological process term/terms, number of essential genes, number of genes, and the names of member genes.
(0.05 MB XLS)

Table S3 Enrichment of ECOBIM and non-ECOBIM COBIM nodes for GO subnetworks in the LC, HC, TAP-MS, BAYESIAN, and Y2H networks. For each GO subnetwork that contributed at least one ECOBIM the fraction of essential proteins among the subnetwork nodes, subnetwork ECOBIM nodes and subnetwork non-ECOBIM COBIM nodes is shown.
(0.15 MB XLS)

Table S4 The parameters of the essential protein interaction model. We use three strategies to estimate the parameters, α and β, of the essential protein interaction model: the network simulation as described in the original paper (simulation), line fitting to points for as described in the original paper (line fitting), and weighted line fitting to points for all values of *k* (weighted line fitting).
(0.03 MB DOC)

Table S5 The number of COBIM and ECOBIMs nodes as a function of the parameter . The number of nodes that belong to one or more COBIMs (ECOBIMs) depends on the value of the parameter *k*. For small values of k the COBIMs (ECOBIMs) output by our algorithm are larger than the COBIMs (ECOBIMs) identified for bigger values of k and therefore contain more network nodes. Here the exact dependency is shown for a range of parameter values. For each protein interaction network the fraction of network nodes that are members of one or more COBIMs (ECOBIMs) is shown. For each network we selected a value of k that results in approximately 25% of network nodes being the members of COBIMs; the resulting fractions are shown in bold.
(0.03 MB DOC)
