# Supplementary material for: Why Do Hubs in the Yeast Protein Interaction Network Tend To Be Essential: Reexamining the Connection between the Network Topology and Essentiality
Source: PLoS Comput Biol. 2008 Aug 1;4(8):e1000140. doi: 10.1371/journal.pcbi.1000140 (PMC2467474; doi:10.1371/journal.pcbi.1000140)
Supplement: Figure S1 — Membership in COBIMs. The amount of overlap among COBIMs is quantified by showing the fraction of nodes that are members of several COBIMs. (0.05 MB DOC) [file pcbi.1000140.s001.doc]

Table S1 - Using network integrity measures to evaluate the effect of the removal of hubs and equivalent number of the most central nodes according to other centrality measures

Two network integrity measures, shortest-path integrity and edge-disjoint paths integrity, are used to quantify the impact of the removal of hubs and equivalent number of the most central nodes according to other centrality measures on the network connectivity. An integrity measure maps a set of nodes S to a value between 0 and 1, with the value of 0 being assigned when the removal of S completely disrupts the communication and the value of 1 being assigned when it causes no disruption. We also show the impact of node removal in random order (rand). These values for the BAYESIAN network are not available (shown as NA), as their computation is computationally demanding.

|  | shortest-path integrity | | | | | |
| --- | --- | --- | --- | --- | --- | --- |
|  | dc | ec | sc | spbc | cfc | rand |
| DIP CORE | 3.31e-03 | 3.03e-01 | 2.14e-01 | 9.39e-03 | 2.19e-03 | 7.88e-01 |
| LC | 1.11e-02 | 3.92e-01 | 3.87e-01 | 7.47e-02 | 3.24e-03 | 8.43e-01 |
| HC | 1.36e-01 | 5.67e-01 | 5.91e-01 | 6.02e-02 | 5.23e-03 | 8.24e-01 |
| TAP-MS | 4.45e-01 | 6.51e-01 | 6.51e-01 | 5.34e-02 | 3.30e-02 | 8.07e-01 |
| BAYESIAN | 1.81e-01 | 6.22e-01 | 6.05e-01 | 9.56e-02 | 5.19e-03 | NA |
| Y2H | 1.55e-03 | 6.39e-02 | 1.81e-02 | 2.08e-03 | 3.20e-03 | 4.14e-01 |

|  | edge-disjoint paths integrity | | | | | |
| --- | --- | --- | --- | --- | --- | --- |
|  | dc | ec | sc | spbc | cfc | rand |
| DIP CORE | 2.23e-03 | 3.15e-01 | 2.10e-01 | 7.08e-03 | 1.68e-03 | 6.89e-01 |
| LC | 1.08e-02 | 3.82e-01 | 3.75e-01 | 8.19e-02 | 2.34e-03 | 7.43e-01 |
| HC | 1.41e-01 | 5.33e-01 | 5.58e-01 | 6.65e-02 | 4.03e-03 | 7.21e-01 |
| TAP-MS | 3.21e-01 | 5.49e-01 | 5.49e-01 | 7.21e-02 | 5.11e-02 | 7.39e-01 |
| BAYESIAN | 1.98e-01 | 5.77e-01 | 5.67e-01 | 1.05e-01 | 1.86e-02 | NA |
| Y2H | 1.29e-03 | 5.66e-02 | 1.57e-02 | 1.78e-03 | 2.75e-03 | 4.04e-01 |
