# Supplementary material for: Why Do Hubs in the Yeast Protein Interaction Network Tend To Be Essential: Reexamining the Connection between the Network Topology and Essentiality
Source: PLoS Comput Biol. 2008 Aug 1;4(8):e1000140. doi: 10.1371/journal.pcbi.1000140 (PMC2467474; doi:10.1371/journal.pcbi.1000140)

Figure S2 – Membership in COBIMs

The amount of overlap among COBIMs is quantified by showing the fraction of nodes that are members of several COBIMs.


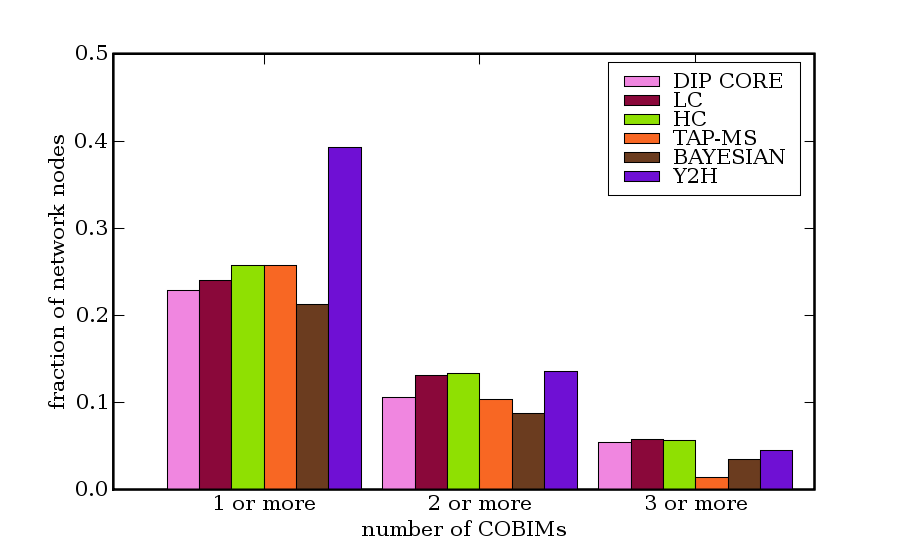

Supplement: Table S1 — Using network integrity measures to evaluate the effect of the removal of hubs and equivalent number of the most central nodes according to other centrality measures (0.04 MB DOC) [file pcbi.1000140.s002.doc]
