# Supplementary material for: Why Do Hubs in the Yeast Protein Interaction Network Tend To Be Essential: Reexamining the Connection between the Network Topology and Essentiality
Source: PLoS Comput Biol. 2008 Aug 1;4(8):e1000140. doi: 10.1371/journal.pcbi.1000140 (PMC2467474; doi:10.1371/journal.pcbi.1000140)
Supplement: Table S4 — The parameters of the essential protein interaction model. We use three strategies to estimate the parameters, α and β, of the essential protein interaction model: the network simulation as described in the original paper (simulation), line fitting to points for as described in the original paper (line fitting), and weighted line fitting to points for all values of k (weighted line fitting). (0.03 MB DOC) [file pcbi.1000140.s005.doc]

Table S5 - The parameters of the essential protein interaction model

We use three strategies to estimate the parameters, α and β, of the essential protein interaction model: the network simulation as described in the original paper (simulation), line fitting to points $(\log(1-P_{E}),k)$ for $k\leq k_{0}$ as described in the original paper (line fitting), and weighted line fitting to points $(\log(1-P_{E}),k)$for all values of k (weighted line fitting).

|  | simulation | | line fitting | | weighted line fitting | |
| --- | --- | --- | --- | --- | --- | --- |
|  | α | β | α | β | α | β |
| DIP CORE | 0.0649 | 0.0816 | 0.0386 | 0.2027 | 0.0278 | 0.2109 |
| LC | 0.0518 | 0.0262 | 0.0376 | 0.1341 | 0.0145 | 0.2068 |
| HC | 0.0662 | 0.0044 | 0.0359 | 0.1833 | 0.0395 | 0.1255 |
| Y2H | 0.0179 | 0.2252 | 0.0696 | 0.0900 | 0.0116 | 0.2149 |
