# Supplementary material for: Why Do Hubs in the Yeast Protein Interaction Network Tend To Be Essential: Reexamining the Connection between the Network Topology and Essentiality
Source: PLoS Comput Biol. 2008 Aug 1;4(8):e1000140. doi: 10.1371/journal.pcbi.1000140 (PMC2467474; doi:10.1371/journal.pcbi.1000140)
Supplement: Table S5 — The number of COBIM and ECOBIMs nodes as a function of the parameter . The number of nodes that belong to one or more COBIMs (ECOBIMs) depends on the value of the parameter k. For small values of k the COBIMs (ECOBIMs) output by our algorithm are larger than the COBIMs (ECOBIMs) identified for bigger values of k and therefore contain more network nodes. Here the exact dependency is shown for a range of parameter values. For each protein interaction network the fraction of network nodes that are members of one or more COBIMs (ECOBIMs) is shown. For each network we selected a value of k that results in approximately 25% of network nodes being the members of COBIMs; the resulting fractions are shown in bold. (0.03 MB DOC) [file pcbi.1000140.s006.doc]

Table S6 – The number of COBIM and ECOBIMs nodes as a function of the parameter k

The number of nodes that belong to one or more COBIMs (ECOBIMs) depends on the value of the parameter k. For small values of k the COBIMs (ECOBIMs) output by our algorithm are larger than the COBIMs (ECOBIMs) identified for bigger values of k and therefore contain more network nodes. Here the exact dependency is shown for a range of parameter values. For each protein interaction network the fraction of network nodes that are members of one or more COBIMs (ECOBIMs) is shown. For each network we selected a value of k that results in approximately 25% of network nodes being the members of COBIMs; the resulting fractions are shown in bold.

|  | k=1 | k=2 | k=3 | k=4 | k=5 | ... | k=11 | k=12 |
| --- | --- | --- | --- | --- | --- | --- | --- | --- |
| DIP CORE |  | 0.43 (0.24) | **0.23 (0.13)** | 0.12 (0.07) |  |  |  |  |
| LC |  | 0.50 (0.33) | 0.35 (0.21) | **0.24 (0.14)** | 0.17 (0.10) |  |  |  |
| HC |  | 0.54 (0.37) | 0.37 (0.24) | **0.26 (0.16)** | 0.19 (0.13) |  |  |  |
| TAP-MS |  | 0.58 (0.32) | 0.50 (0.28) | 0.43 (0.24) | 0.39 (0.22) |  | **0.26 (0.15)** | 0.24 (0.14) |
| BAYESIAN |  | 0.36 (0.18) | 0.27 (0.16) | **0.21 (0.13)** | 0.18 (0.11) |  |  |  |
| Y2H | **0.39 (0.14)** | 0.06 (0.01) | 0.02 (0.01) |  |  |  |  |  |
